# Crystal structure of a (carb­oxy­meth­yl)tri­ethyl­aza­nium bromide–2-(tri­ethyl­aza­n­ium­yl)acetate (1/1) hydrogen-bonded dimer

**DOI:** 10.1107/S2056989023006850

**Published:** 2023-08-10

**Authors:** Faith M. Carlson, Richard J. Staples, Shannon M. Biros

**Affiliations:** a Department of Chemistry, Grand Valley State University, Allendale, MI 49401, USA; b Center for Crystallographic Research, Department of Chemistry, Michigan State University, East Lansing, MI 48824, USA; Vienna University of Technology, Austria

**Keywords:** crystal structure, asymmetric hydrogen bond, bromide salt, inter­molecular O—H⋯O hydrogen bond, intra­molecular C—H⋯O hydrogen bond

## Abstract

The title compound, C_8_H_18_NO_2_^+^·Br^−^·C_8_H_17_NO_2_, crystallizes as the bromide salt of a 50:50 mixture of (tri­ethyl­azaniumyl)­carb­oxy­lic acid and the zwitterionic (tri­ethyl­azaniumyl)­carboxyl­ate. The two organic entities are linked by a half-occupied bridging carb­oxy­lic acid hydrogen atom that is hydrogen-bonded to the carboxyl­ate group of the second mol­ecule. The tetra­lkyl­ammonium group adopts a nearly perfect tetra­hedral shape around the nitro­gen atom with bond lengths that agree with known values. The carb­oxy­lic acid/carboxyl­ate group is oriented *anti* to one of the ethyl groups on the ammonium group, and the carbonyl oxygen atom is engaged in intra­molecular C—H⋯O hydrogen bonds.

## Abstract 2

The crystal structure of a hydrogen-bonded dimer of (tri­ethyl­azaniumyl)­acetic acid and (tri­ethyl­azaniumyl)­acetate bromide features a carb­oxy­lic acid hydrogen atom that is engaged in an asymmetric hydrogen bond with the carboxyl­ate oxygen. The crystal also features intra­molecular C—H hydrogen bonds and a layer of bromide ions that is surrounded by alkyl groups.

## 1. Chemical context

**Figure 1:**
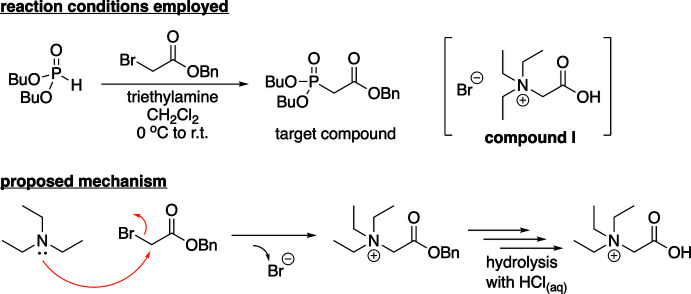
(top) The reaction carried out in this work, along with structures of the target *β*-carbonyl­phospho­nate and the title compound **I**. (bottom) A proposed mechanism for the formation of the title compound.

The *β*-carbonyl­phospho­nate moiety is commonly used as a reagent in the Horner–Wadsworth–Emmons reaction (Horner *et al.*, 1958 ▸; Wadsworth & Emmons, 1961 ▸; Bisceglia & Orelli, 2015 ▸). These mol­ecules are reacted with aldehydes or ketones to prepare *α,β*-unsaturated esters, where a preference for the *Z*-configuration of the alkene group is often observed. When the phospho­nate group is replaced with a phosphine oxide, these sets of compounds have found use as ligands and extraction agents for *f*-elements (Babecki *et al.*, 1989 ▸, 1990 ▸, 1992 ▸). Our research group has also characterized the ability of these types of compounds to sensitize the luminescence of lanthanide ions (Leach *et al.*, 2017 ▸; Sartain *et al.*, 2015 ▸). To this end, our group has been working to develop a synthetic route to the target compound shown in Fig. 1 ▸, following the procedure reported by Ando (1999 ▸). The title compound was an undesired byproduct of this reaction, and serendipitously crystallized from the aqueous washings upon standing. A proposed mechanism for the formation of the title compound **I** is also shown in Fig. 1 ▸. 
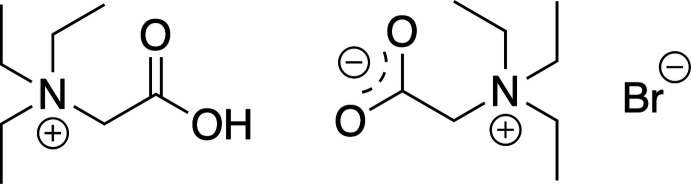


## 2. Structural commentary

**Figure 2:**
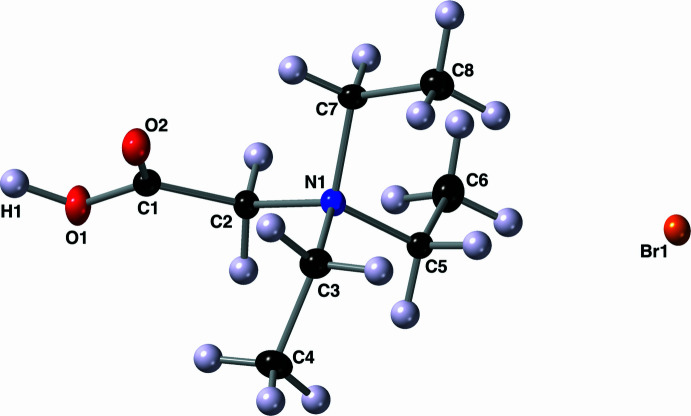
The mol­ecular structure of compound **I**, with the atom-labeling scheme. Displacement ellipsoids are drawn at the 50% probability level using standard CPK colors. Atom H1 shows half-occupancy.

**Figure 3:**
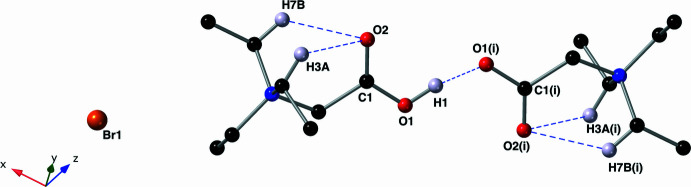
A depiction of the hydrogen-bonding inter­actions present in the crystal of compound **I** using a ball-and-stick model with standard CPK colors. Hydrogen-bonding inter­actions are depicted with blue dashed lines and all hydrogen atoms not involved in a hydrogen bond are not shown for clarity.

The title salt crystallizes as a 50:50 mixture of the ammonium carboxyl­ate zwitterion and the ammonium bromide. The mol­ecular entities of this compound are shown in Fig. 2 ▸ along with the atom-numbering scheme. The asymmetric unit is composed of all of the atoms shown in Fig. 2 ▸ where the carb­oxy­lic acid hydrogen atom H1 has a 0.50 occupancy, and the Br^−^ anion is located on a twofold rotation axis (Wyckoff position 4*e*) of space group *I*2/*a*. The ammonium group has C—N bond lengths ranging from 1.514 (3) to 1.526 (3) Å with a nearly perfect tetra­hedral arrangement of alkyl groups around the nitro­gen atom with a *τ*_4_ descriptor for fourfold coordin­ation of 0.97 (where 0.00 = square-planar, 0.85 = trigonal–pyramidal, and 1.00 = tetra­hedral; Yang *et al.*, 2007 ▸). The carb­oxy­lic acid group has C—O bond lengths of 1.286 (3) and 1.224 (3) Å. When the mol­ecule is viewed down the C2—N1 bond the groups adopt a staggered conformation with the carb­oxy­lic acid group being *anti* to the C5–C6 ethyl group. The torsion angle between these two groups (C1—C2—N1—C5) is 168.8 (2)°. Two intra­molecular C—H⋯O hydrogen bonds are present between the carbonyl oxygen atom O2 and hydrogen atoms H3*A* and H7*B* of the *gauche* alkyl groups (Table 1 ▸, Fig. 3 ▸).

## 3. Supra­molecular features

**Figure 4:**
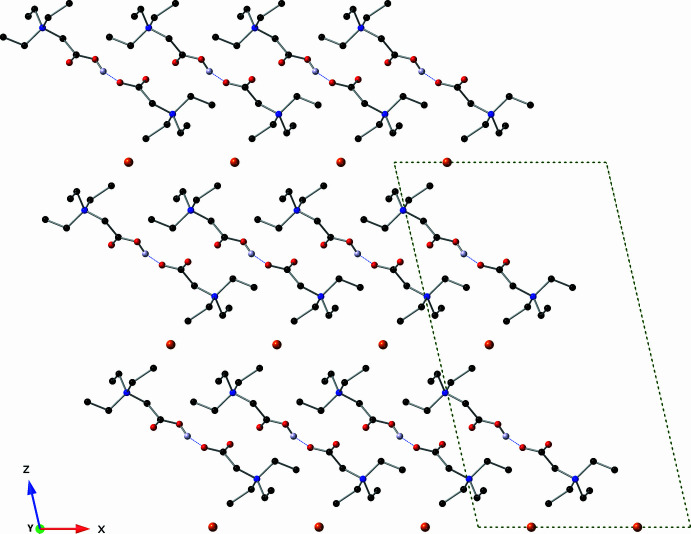
A view of the crystal structure down the *b* axis showing a cross section of the layers of bromide ions. This figure was drawn with a ball-and-stick model using standard CPK colors. Only one position of hydrogen atom H1 is shown, and all other hydrogen atoms have been omitted for clarity.

Mol­ecules of the title compound exist as hydrogen-bonded dimers in the solid state. The carb­oxy­lic acid hydrogen atom H1 is a half-occupied bridging hydrogen atom (Fábry, 2018 ▸), and within this dimer it is either bonded to oxygen atom O1 or to its symmetry derived counterpart O1^i^ [symmetry code: (i) –*x* + 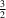 , –*y* + 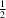 , –*z* + 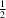 ; Fig. 3 ▸]. When this atom H1 is covalently bonded to O1, it is engaged in a very strong asymmetric hydrogen bond with the symmetry-derived oxygen atom O1^i^ (Table 1 ▸). The bromide counter-ions are located away from the carboxyl­ate/carb­oxy­lic acid sites and occupy a layer that lies parallel to the *ab* plane. These layers are bordered by the ethyl chains of the ammonium groups (Fig. 4 ▸).

## 4. Database survey

A search of the Cambridge Structural Database (CSD version 5.43, Jun. 2022; Groom *et al.*, 2016 ▸) for structures with a hydrogen atom shared between two carboxyl­ate sites resulted in 274 hits. One of the simplest structures in this set is that of ammonium di­acetate (ACAMAC; Nahringbauer, 1969 ▸). The structures ALUNIE (Dega-Szafran *et al.*, 2003 ▸) and CIVKUQ (Ghaza­ryan *et al.*, 2018 ▸) are similar to the title compound with either a piperidinium ring or a tri­methyl­ammonium group, respectively, in the place of the tri­ethyl­ammonium groups. Both compounds were isolated with a halide counter-ion: ALUNIE was isolated with one chloride anion and CIVKUQ was isolated as the iodide salt.

## 5. Synthesis and crystallization

Dibutyl phosphite (1.4 ml, 1.4 g, 7.17 mmol) was added *via* syringe to a two-necked 25 ml round-bottom flask under an atmosphere of nitro­gen. The reagent was dissolved in 7.0 ml of di­chloro­methane and the flask was placed in an ice–water bath. Benzyl bromo­acetate (1.1 ml, 1.6 g, 6.94 mmol) and tri­ethyl­amine (1.4 ml, 1.0 g, 10.0 mmol) were added and the reaction mixture was stirred for 15 minutes in the ice bath followed by one hour at room temperature. Water (10 ml) was added to the reaction, and the aqueous layer was washed with ethyl acetate (3×10 ml). The organic extracts were combined and washed with 1 *M* HCl (3×10 ml) and brine (1×10 ml), then dried over MgSO_4_. The title compound crystallized serendipitously from the combined aqueous washings after standing for *ca* three days.

## 6. Refinement

Crystal data, data collection and structure refinement details are summarized in Table 2 ▸. All hydrogen atoms bonded to carbon atoms were placed in calculated positions and refined as riding: C—H = 0.95 – 1.00 Å with *U*_iso_(H) = 1.2*U*_eq_(C) for methyl­ene hydrogen atoms and *U*_iso_(H) = 1.5*U*_eq_(C) for the hydrogen atoms of the methyl groups. The carb­oxy­lic acid hydrogen atom H1 was located using electron-density difference maps. The position of this hydrogen atom was fixed and the occupancy constrained to 0.5. Its isotropic displacement parameter was refined as suggested by Fábry (2018 ▸).

## Supplementary Material

Crystal structure: contains datablock(s) I. DOI: 10.1107/S2056989023006850/wm5689sup1.cif

Structure factors: contains datablock(s) I. DOI: 10.1107/S2056989023006850/wm5689Isup2.hkl

Supporting information file. DOI: 10.1107/S2056989023006850/wm5689Isup3.cml

CCDC reference: 2286618

Additional supporting information:  crystallographic information; 3D view; checkCIF report

## Figures and Tables

**Table 1:** Hydrogen-bond geometry (Å, °)

*D*—H⋯*A*	*D*—H	H⋯*A*	*D*⋯*A*	*D*—H⋯*A*
O1—H1⋯O1^i^	0.97	1.50	2.457 (4)	167
C3—H3*A*⋯O2	0.99	2.39	2.969 (3)	116
C7—H7*B*⋯O2	0.99	2.39	3.068 (3)	125

Symmetry code: (i) 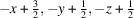 .

**Table 2:** Experimental details

Crystal data	
Chemical formula	C_8_H_18_NO_2_^+^·Br^−^·C_8_H_17_NO_2_
*M*_r_	399.37
Crystal system, space group	Monoclinic, *I*2/*a*
Temperature (K)	100
*a*, *b*, *c* (Å)	12.6692 (4), 7.0967 (3), 22.3413 (9)
β (°)	103.022 (4)
*V* (Å^3^)	1957.04 (13)
*Z*	4
Radiation type	Cu *K*α
μ (mm^−1^)	3.03
Crystal size (mm)	0.42 × 0.13 × 0.02

Data collection	
Diffractometer	XtaLAB Synergy, Dualflex, HyPix
Absorption correction	Gaussian (*CrysAlis PRO*; Oxford Diffraction, 2006 ▸)
*T*_min_, *T*_max_	0.568, 1.000
No. of measured, independent and observed [*I* > 2σ(*I*)] reflections	6198, 1998, 1785
*R*_int_	0.052
(sin θ/λ)_max_ (Å^−1^)	0.639

Refinement	
*R*[*F* ^2^ > 2σ(*F* ^2^)], *wR*(*F* ^2^), *S*	0.040, 0.109, 1.08
No. of reflections	1998
No. of parameters	109
H-atom treatment	H atoms treated by a mixture of independent and constrained refinement
Δρ_max_, Δρ_min_ (e Å^−3^)	0.89, −0.42

Computer programs: *CrysAlis PRO* (Oxford Diffraction, 2006 ▸), *SHELXT* (Sheldrick, 2015*a* ▸), *SHELXL* (Sheldrick, 2015*b* ▸), *CrystalMaker* (Palmer, 2007 ▸), and *OLEX2* (Dolomanov *et al.*, 2009 ▸; Bourhis *et al.*, 2015 ▸).

## supplementary crystallographic information

### Crystal data

**Table:**

C_8_H_18_NO_2_^+^·Br^−^·C_8_H_17_NO_2_	*F*(000) = 848
*M**_r_* = 399.37	*D*_x_ = 1.355 Mg m^−^^3^
Monoclinic, *I*2/*a*	Cu *K*α radiation, λ = 1.54184 Å
*a* = 12.6692 (4) Å	Cell parameters from 3269 reflections
*b* = 7.0967 (3) Å	θ = 6.6–79.4°
*c* = 22.3413 (9) Å	µ = 3.03 mm^−^^1^
β = 103.022 (4)°	*T* = 100 K
*V* = 1957.04 (13) Å^3^	Plate, colourless
*Z* = 4	0.42 × 0.13 × 0.02 mm

### Data collection

**Table:**

XtaLAB Synergy, Dualflex, HyPix diffractometer	1785 reflections with *I* > 2σ(*I*)
Detector resolution: 10.0000 pixels mm^-1^	*R*_int_ = 0.052
ω scans	θ_max_ = 80.0°, θ_min_ = 4.1°
Absorption correction: gaussian (CrysAlisPro; Oxford Diffraction, 2006)	*h* = −11→16
*T*_min_ = 0.568, *T*_max_ = 1.000	*k* = −8→8
6198 measured reflections	*l* = −28→25
1998 independent reflections	

### Refinement

**Table:**

Refinement on *F*^2^	0 restraints
Least-squares matrix: full	Hydrogen site location: mixed
*R*[*F*^2^ > 2σ(*F*^2^)] = 0.040	H atoms treated by a mixture of independent and constrained refinement
*wR*(*F*^2^) = 0.109	*w* = 1/[σ^2^(*F*_o_^2^) + (0.0638*P*)^2^ + 1.4873*P*] where *P* = (*F*_o_^2^ + 2*F*_c_^2^)/3
*S* = 1.08	(Δ/σ)_max_ < 0.001
1998 reflections	Δρ_max_ = 0.89 e Å^−^^3^
109 parameters	Δρ_min_ = −0.42 e Å^−^^3^

### Special details

**Table:**

Geometry. All esds (except the esd in the dihedral angle between two l.s. planes) are estimated using the full covariance matrix. The cell esds are taken into account individually in the estimation of esds in distances, angles and torsion angles; correlations between esds in cell parameters are only used when they are defined by crystal symmetry. An approximate (isotropic) treatment of cell esds is used for estimating esds involving l.s. planes.

### Fractional atomic coordinates and isotropic or equivalent isotropic displacement parameters (Å^2^)

**Table:**

	*x*	*y*	*z*	*U*_iso_*/*U*_eq_	Occ. (<1)
Br1	0.250000	0.39582 (5)	0.500000	0.01824 (16)	
O1	0.70415 (15)	0.3474 (3)	0.28191 (9)	0.0250 (4)	
H1	0.734798	0.280169	0.252098	0.07 (3)*	0.5
O2	0.57904 (15)	0.1209 (3)	0.27312 (9)	0.0235 (4)	
N1	0.49061 (15)	0.3014 (3)	0.36825 (9)	0.0146 (4)	
C1	0.61984 (19)	0.2698 (4)	0.29428 (11)	0.0181 (5)	
C2	0.57456 (19)	0.3923 (3)	0.33887 (12)	0.0155 (5)	
H2A	0.635817	0.434190	0.372016	0.019*	
H2B	0.542009	0.506334	0.316589	0.019*	
C3	0.5279 (2)	0.1112 (3)	0.39641 (12)	0.0187 (5)	
H3A	0.522278	0.017911	0.362887	0.022*	
H3B	0.477980	0.070888	0.422252	0.022*	
C4	0.6431 (2)	0.1076 (4)	0.43539 (14)	0.0242 (6)	
H4A	0.694224	0.132344	0.409276	0.036*	
H4B	0.658203	−0.016535	0.454658	0.036*	
H4C	0.651144	0.204548	0.467320	0.036*	
C5	0.47364 (19)	0.4306 (4)	0.41955 (11)	0.0168 (5)	
H5A	0.419692	0.371758	0.439611	0.020*	
H5B	0.542724	0.439647	0.450705	0.020*	
C6	0.4358 (2)	0.6278 (4)	0.39983 (14)	0.0255 (6)	
H6A	0.369669	0.621107	0.367223	0.038*	
H6B	0.492434	0.693461	0.384459	0.038*	
H6C	0.420881	0.696650	0.435042	0.038*	
C7	0.38591 (18)	0.2757 (4)	0.31950 (11)	0.0170 (5)	
H7A	0.360683	0.400804	0.302461	0.020*	
H7B	0.401558	0.198822	0.285550	0.020*	
C8	0.2954 (2)	0.1824 (4)	0.34289 (13)	0.0242 (6)	
H8A	0.228952	0.182944	0.310402	0.036*	
H8B	0.283092	0.251692	0.378634	0.036*	
H8C	0.315681	0.052079	0.354755	0.036*	

### Atomic displacement parameters (Å^2^)

**Table:**

	*U*^11^	*U*^22^	*U*^33^	*U*^12^	*U*^13^	*U*^23^
Br1	0.0155 (2)	0.0211 (2)	0.0191 (2)	0.000	0.00614 (14)	0.000
O1	0.0209 (9)	0.0313 (10)	0.0264 (10)	−0.0062 (8)	0.0132 (8)	−0.0083 (8)
O2	0.0214 (9)	0.0270 (10)	0.0242 (10)	−0.0037 (7)	0.0091 (8)	−0.0089 (7)
N1	0.0128 (8)	0.0161 (10)	0.0154 (10)	−0.0009 (7)	0.0043 (8)	0.0001 (8)
C1	0.0161 (10)	0.0229 (13)	0.0149 (12)	0.0010 (9)	0.0029 (9)	−0.0013 (9)
C2	0.0138 (10)	0.0171 (12)	0.0162 (12)	−0.0029 (8)	0.0050 (9)	−0.0002 (9)
C3	0.0212 (12)	0.0158 (12)	0.0186 (12)	0.0001 (8)	0.0035 (10)	0.0024 (9)
C4	0.0213 (12)	0.0252 (14)	0.0233 (14)	0.0057 (10)	−0.0010 (10)	0.0019 (10)
C5	0.0151 (10)	0.0214 (12)	0.0140 (11)	0.0015 (9)	0.0033 (9)	−0.0002 (9)
C6	0.0315 (14)	0.0214 (14)	0.0255 (15)	0.0075 (10)	0.0107 (12)	0.0003 (10)
C7	0.0136 (9)	0.0218 (12)	0.0145 (11)	−0.0025 (9)	0.0008 (9)	−0.0003 (9)
C8	0.0159 (11)	0.0331 (15)	0.0231 (13)	−0.0068 (10)	0.0032 (10)	−0.0005 (11)

### Geometric parameters (Å, º)

**Table:**

O1—H1	0.9686	C4—H4B	0.9800
O1—C1	1.286 (3)	C4—H4C	0.9800
O2—C1	1.224 (3)	C5—H5A	0.9900
N1—C2	1.514 (3)	C5—H5B	0.9900
N1—C3	1.519 (3)	C5—C6	1.512 (4)
N1—C5	1.520 (3)	C6—H6A	0.9800
N1—C7	1.526 (3)	C6—H6B	0.9800
C1—C2	1.528 (3)	C6—H6C	0.9800
C2—H2A	0.9900	C7—H7A	0.9900
C2—H2B	0.9900	C7—H7B	0.9900
C3—H3A	0.9900	C7—C8	1.515 (3)
C3—H3B	0.9900	C8—H8A	0.9800
C3—C4	1.522 (4)	C8—H8B	0.9800
C4—H4A	0.9800	C8—H8C	0.9800
			
C1—O1—H1	114.7	H4A—C4—H4C	109.5
C2—N1—C3	112.00 (18)	H4B—C4—H4C	109.5
C2—N1—C5	107.61 (18)	N1—C5—H5A	108.4
C2—N1—C7	108.91 (18)	N1—C5—H5B	108.4
C3—N1—C5	107.89 (19)	H5A—C5—H5B	107.5
C3—N1—C7	109.20 (19)	C6—C5—N1	115.3 (2)
C5—N1—C7	111.24 (17)	C6—C5—H5A	108.4
O1—C1—C2	110.4 (2)	C6—C5—H5B	108.4
O2—C1—O1	125.8 (2)	C5—C6—H6A	109.5
O2—C1—C2	123.7 (2)	C5—C6—H6B	109.5
N1—C2—C1	116.35 (19)	C5—C6—H6C	109.5
N1—C2—H2A	108.2	H6A—C6—H6B	109.5
N1—C2—H2B	108.2	H6A—C6—H6C	109.5
C1—C2—H2A	108.2	H6B—C6—H6C	109.5
C1—C2—H2B	108.2	N1—C7—H7A	108.7
H2A—C2—H2B	107.4	N1—C7—H7B	108.7
N1—C3—H3A	108.5	H7A—C7—H7B	107.6
N1—C3—H3B	108.5	C8—C7—N1	114.2 (2)
N1—C3—C4	114.9 (2)	C8—C7—H7A	108.7
H3A—C3—H3B	107.5	C8—C7—H7B	108.7
C4—C3—H3A	108.5	C7—C8—H8A	109.5
C4—C3—H3B	108.5	C7—C8—H8B	109.5
C3—C4—H4A	109.5	C7—C8—H8C	109.5
C3—C4—H4B	109.5	H8A—C8—H8B	109.5
C3—C4—H4C	109.5	H8A—C8—H8C	109.5
H4A—C4—H4B	109.5	H8B—C8—H8C	109.5
			
O1—C1—C2—N1	−167.3 (2)	C3—N1—C7—C8	56.4 (3)
O2—C1—C2—N1	13.8 (4)	C5—N1—C2—C1	168.8 (2)
C2—N1—C3—C4	46.5 (3)	C5—N1—C3—C4	−71.8 (3)
C2—N1—C5—C6	59.1 (3)	C5—N1—C7—C8	−62.6 (3)
C2—N1—C7—C8	179.0 (2)	C7—N1—C2—C1	−70.5 (3)
C3—N1—C2—C1	50.4 (3)	C7—N1—C3—C4	167.2 (2)
C3—N1—C5—C6	−179.9 (2)	C7—N1—C5—C6	−60.1 (3)

### Hydrogen-bond geometry (Å, º)

**Table:**

*D*—H···*A*	*D*—H	H···*A*	*D*···*A*	*D*—H···*A*
O1—H1···O1^i^	0.97	1.50	2.457 (4)	167
C3—H3*A*···O2	0.99	2.39	2.969 (3)	116
C7—H7*B*···O2	0.99	2.39	3.068 (3)	125

Symmetry code: (i) −*x*+3/2, −*y*+1/2, −*z*+1/2.
